# Investigation of the influence of fluid dynamics on thrombus growth at the interface between a connector and tube

**DOI:** 10.1007/s10047-017-0973-6

**Published:** 2017-07-28

**Authors:** Yuki Matsuhashi, Kei Sameshima, Yoshiki Yamamoto, Mitsuo Umezu, Kiyotaka Iwasaki

**Affiliations:** 1 Department of Integrative Bioscience and Biomedical Engineering, Graduate School of Advanced Science and Engineering, Waseda University, Shinjuku, Tokyo Japan; 2 Department of Modern Mechanical Engineering, Graduate School of Creative Science and Engineering, Waseda University, Shinjuku, Tokyo Japan; 3 Cooperative Major in Advanced Biomedical Sciences, Graduate School of Advanced Science and Engineering, Waseda University, Shinjuku, Tokyo Japan

**Keywords:** Thrombus formation, Real-time visualization method, Optical coherence tomography, Stagnant flow, Reattachment point of flow

## Abstract

Thrombus formation at the interface between connectors and tubes is a potential risk factor for complications. We investigated time-dependent relationships between formation of thrombus and hemodynamic factors at the interface between connectors and tubes using optical coherence tomography (OCT) under pulsatile flow. A swept-source OCT with the center wavelength of 1330 nm was employed. The sequential process of thrombus formation at the interface of connectors and tubes in the inlet and outlet was investigated. Connectors with and without tapers were tested using identical 50-ml air-contactless circuits. Fresh human blood from healthy volunteers was circulated under pulsatile flow. Thrombus initially formed at the interface between the connector tip and the tube. Geometries of thrombus growth were different between the 2 connectors, and between the inlet and the outlet. Growth of thrombus was observed at the interface between the connectors and tubes over time in 60 min circulation, except at the outlet part of connector without tapers. At the connector without tapers outlet, thrombus propagation length from the connector edge toward the flow downstream was comparable at 10 and 60 min (0.55 ± 0.35 vs. 0.51 ± 0.32 mm, *p* = 0.83). Analysis using particle image velocimetry showed the presence of a flow reattachment point 1.5 mm downstream from the connector edge. These results suggest that the flow reattachment point inhibits downstream thrombus growth. We quantitatively demonstrated sequential thrombus process at the interface between the connectors and tubes under pulsatile flow of human blood using OCT.

## Introduction

Medical devices with blood-contact surfaces, such as extracorporeal membrane oxygenation (ECMO) for cardiopulmonary support of critically ill patients or continuous hemofiltration therapy for renal failure patients, improve the quality of life and expand treatment options [1, 2]. In these medical devices, thrombus formation at the interface between a connector and tube has been reported [3, 4]. Because a thrombus is a potential risk factor for complications, the relationship between thrombus formation and hemodynamic flow has been investigated by many researchers. However, the causal relationship is not yet fully understood. Understanding the relationship between hemodynamic factors influenced by medical device geometry and thrombus formation may lead to development of a novel design to regulate thrombus formation at the interface between connecting parts. Previous studies on thrombus formation have focused on the hemodynamic interactions with biological and biochemical responses of platelets, erythrocytes, and plasma proteins. These studies showed that thrombus growth is closely related to the extent of stagnation and separation under steady flow [5–11]. However, there has been little research on the effect of pulsatile flow on thrombus-related reactions [12, 13], presumably because flow is time-dependent and complex in pulsatile circulation. Furthermore, most previous studies used platelet suspensions in serum or phosphate-buffered saline, and excluded red and/or white blood cells to visualize platelet aggregation phenomena. Therefore, thrombus formation due to the interaction of all blood components may be overlooked [14–17].

The aim of this study was to investigate time-dependent relationships between the formation of thrombus and hemodynamic factors at the interface between connectors and tubes under pulsatile flow using optical coherence tomography (OCT). To investigate the influence of hemodynamic factors on formation of thrombus, connectors with and without tapers were compared.

## Materials and methods

### Real-time visualization system of thrombus formation

**Fig. 1:**
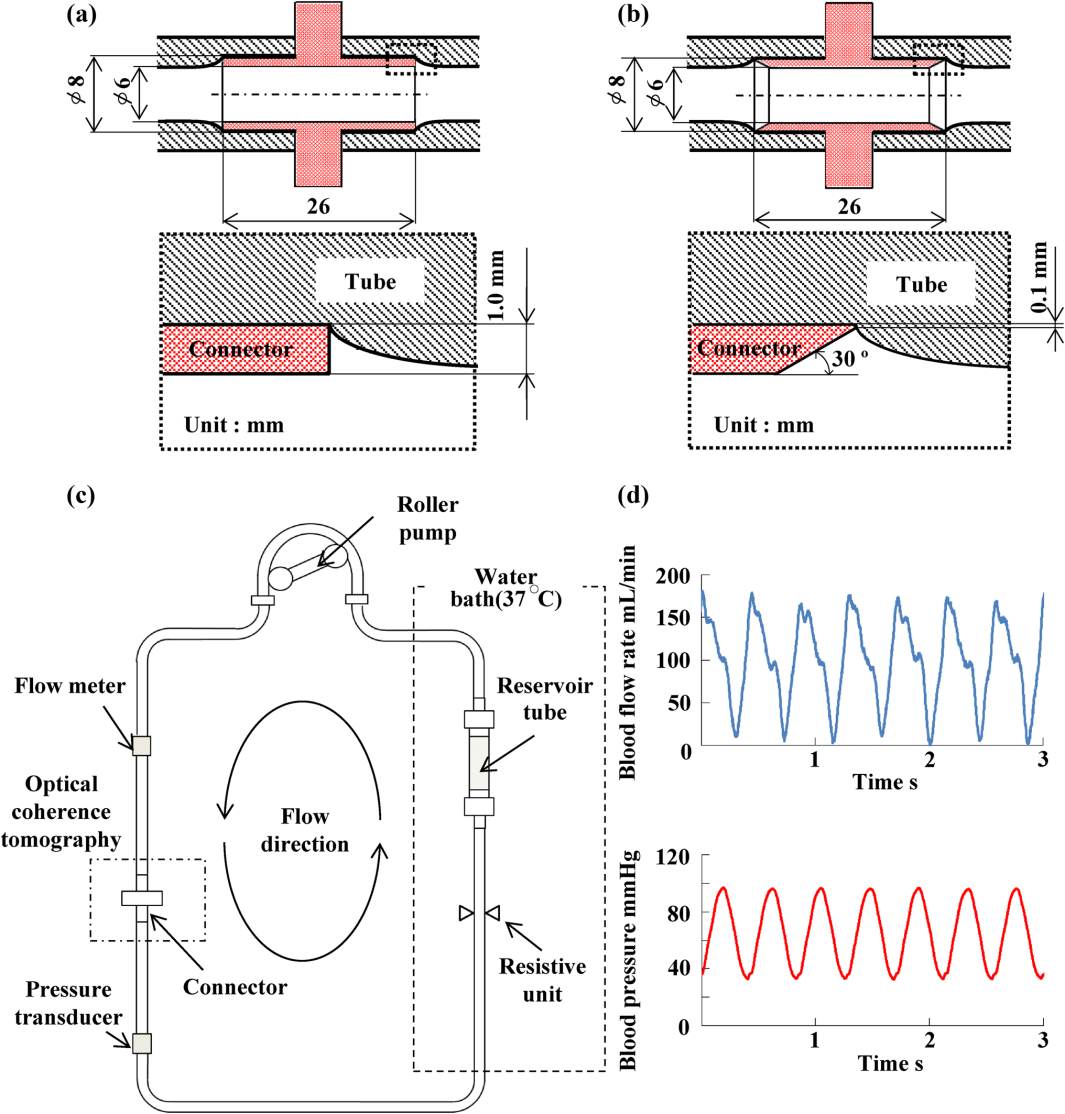
Schematic of a sequential visualization system of thrombus formation using optical coherence tomography. **a** Connector without tapers. **b** Connector with tapers. **c** Schematic of in vitro blood circulation circuit. **d** Blood flow and pressure waveforms

A swept-source OCT (Panasonic Healthcare, Japan) with a center wavelength and maximal energy of 1330 nm and 15 mW was employed to non-invasively examine the sequential process of thrombus formation. For testing, connectors with a 6-mm inner diameter and 8-mm outer diameter, without tapering at either end (Fig. 1a), or with taper (Fig. 1b) were manufactured. The inner and outer diameters of the connectors are similar to those of commercial connectors used in hemofiltration therapies. Due to the manufacturer, edge shapes of commercially available connectors are different. For example, one connector has a 10° taper to the tip and 300 μm width at the tip. Another connector has corner radium of 1 mm and 500 μm width at the tip. Another connector has no taper and 500 μm width at the tip. In this study, two connectors with 30° taper and tip width of 100 μm and without taper and tip width of 1.0 mm were manufactured. These connectors were made of polyurethane (SG7101 AT,SG7101 B, SID Co., Ltd., Saitama, Japan). The connectors were joined to polyvinylchloride (PVC) tubes with an inner diameter of 6 mm (Tygon^®^, ACFJ00007, Saint-Gobain, Tokyo, Japan). The process of thrombus formation at the interface between the connectors and tubes was examined. The connectors and tubes were placed into an air-contactless closed circuit with a total volume of 50 mL. The circuit consisted of a roller pump (Masterflex 07528, Yamato Scientific Co., Ltd., Tokyo, Japan), a compliant reservoir tube made of blood-compatible segmented polyurethane (TM-5, Nipro Co., Ltd, Osaka, Japan), the PVC tubes, and a resistive unit (Hoffman pinchcock, AS ONE, Osaka, Japan) (Fig. 1c). A compliant reservoir tube with an inner diameter of 12 mm and length of 30 mm was made using a dipping method. Blood-contact surfaces of the PVC tubes and connectors were coated twice with a segmented polyurethane (Miractran^®^, Nihon Unipolymer, Tokyo, Japan), and then coated twice with 2-methacryloyloxyethyl phosphorylcholine (MPC) (Lipidure-CM5206, NOF CO. Tokyo, Japan). The coating procedure and manufacture of the compliant reservoir tube were performed in a class 100 clean air room. All components of the circuit were sterilized by ethylene oxide gas before use in the tests.

### Blood sampling and analysis

This study was approved by the ethics committee of Waseda University (2015-212) and performed in accordance with the ethical standards stated in the 1964 Declaration of Helsinki and its later amendments. With informed consent, blood was sampled from 6 healthy volunteers. The blood bags (BB-T040CJ, TERUMO Corp., Tokyo, Japan) held 250 mL, and were coated with MPC twice in the clean air room and sterilized with ethylene oxide gas before use. A 22-G indwelling needle was inserted into a peripheral vein. Then, 250 mL blood was drawn by gravity drainage into the blood bag. To avoid the collection of activated platelets, the first 2 mL of blood was discarded. Blood sampling was performed aseptically. Prior to drawing blood, 0.4 units/mL heparin (Heparin Sodium, Mochida Pharmaceutical Co., Ltd., Tokyo, Japan) was injected into the blood bag to expect the activated clotting time of approximately 150–180 s.

The activated clotting time was measured using a whole blood coagulation system (Hemocron^®^ Response, Accriva Diagnostics, representing ITC and Accumetrics, Edison, NJ, USA) before and after the test.

Blood pH was measured using a portable blood gas analyzer (i-STAT^®^, Abbott Point of Care Inc., Princeton, NJ, USA) with a cartridge (G3+, Abbott Point of Care). The platelet count and the hematocrit were measured using an automated hematology analyzer (Celltac E MEK-7222, Nihon Kohden Corp., Tokyo, Japan) and hematocrit centrifuge (Model 3220, Kubota Corp., Tokyo, Japan). Blood viscosity was measured using a rotational viscometer (TPE-100, Toki Sangyo Co., Ltd., Tokyo, Japan) at 37 °C and the shear rate of 383/s. To avoid coagulation during the measurement of viscosity, blood was sampled to 3.2% sodium citrate blood collection tubes (VP-CA053 K, TERUMO Co., Tokyo, Japan).

### Blood circulation test

Two identical circuits except for 2 connectors with different configurations were prepared. The circuits were filled with identical-source blood. The blood circulation tests were performed for 60 min. Using the roller pump, pulsatile flow was circulated under 27 rpm. The mean blood flow rate and pressure were adjusted to 100 mL/min and 70 mmHg using the roller pump and resistive unit (Fig. 1d). These flow and pressure conditions are prevalent in continuous hemofiltration therapy in Japan. Blood flow rate and pressure were monitored using an ultrasonic blood flow meter (ME4PXL/N, Transonic systems Inc., NY, USA) and pressure transducer (UK-801, Baxter, Irvine, CA, USA). The compliant reservoir tube and part of the PVC tube were placed in a 37 °C water bath to regulate circulating blood temperature. OCT images of the interface between the connecter and the tube were sequentially taken in a vertical direction at 10, 20, 30, 40, 50, and 60 min. Image acquisition was performed at 7 frames/s with 20 μm × 20 μm size. After the circulation tests, blood was immediately removed from the circuit, and the interfaces between the connectors and tubes were rinsed carefully with phosphate-buffered saline 3 times.

### Thrombus extraction method using OCT images

The average of 6 consecutive time-difference images in 1 s just after the start of blood circulation was used to differentiate the still connectors and tubes from circulating blood. The still area of connectors and tubes was defined as the background image. Each of the 6 averaged consecutive time-difference images was analyzed at the start of circulation, and at 10, 20, 30, 40, 50, and 60 min. Otsu’s method [18] was used to calculate the optimal threshold separating the still area and the area of circulating blood. The background image was subtracted from the extracted images to define the area where thrombus formed.

### Visualization of flow field using particle imaging velocimetry

**Fig. 2:**
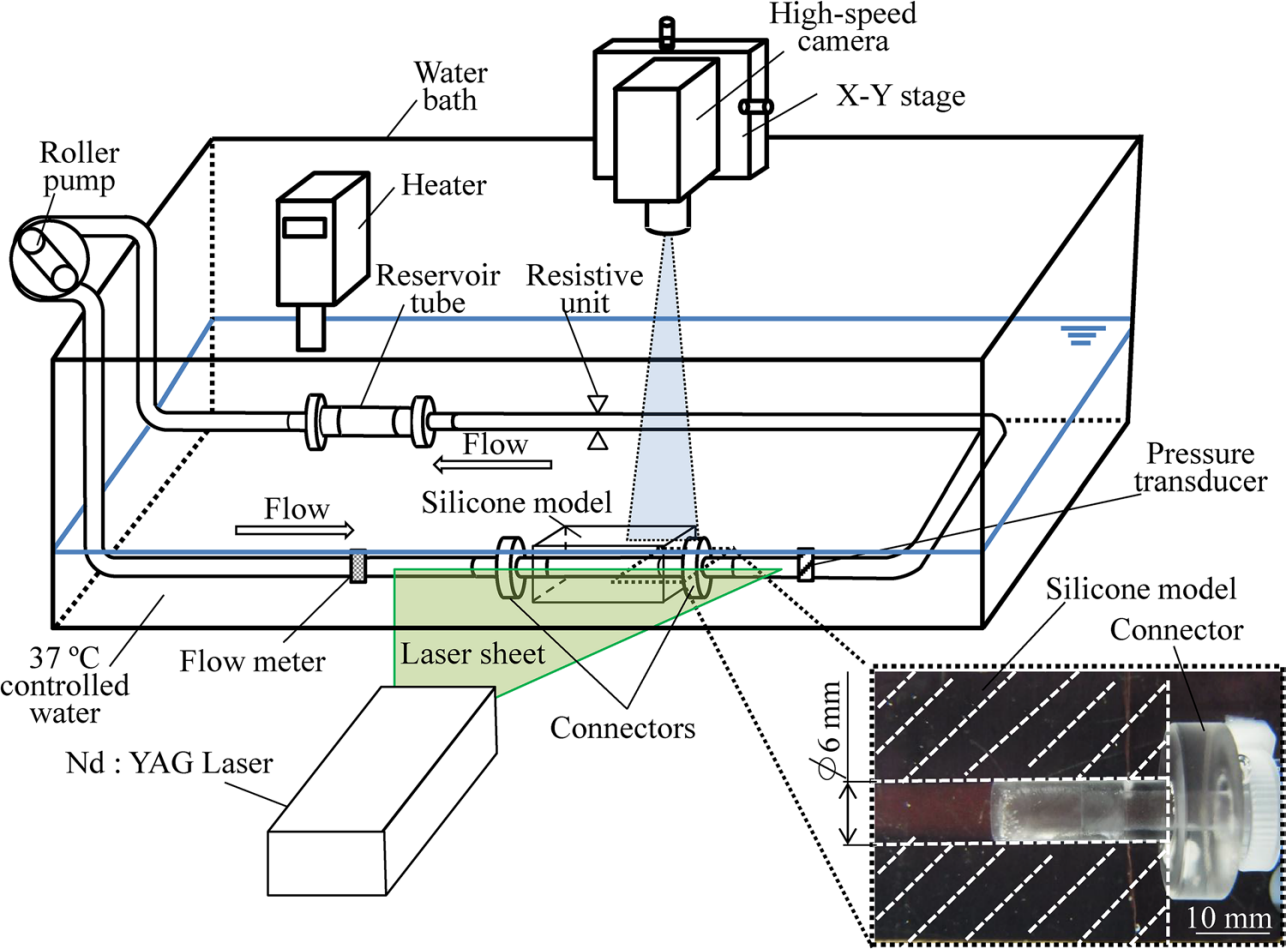
Experimental system for visualizing flow field at the interface between the connectors and tubes using particle image velocimetry

The blood flow field at the interface between the connectors and tubes was examined using fluorescent particle imaging velocimetry (PIV). The circuit components, roller pump, a compliant reservoir tube, the PVC tubes, and a resistive unit were the same as in the blood circulation test. A straight flow tract with an inner diameter of 6 mm was manufactured using a block of silicone (KE-1603,Shin-Etsu Chemical, Tokyo, Japan), as an alternative to the PVC tube, in order to match the refractive index of the tube with that of working fluid as 1.4096 (Fig. 2).

Young’s modulus of the silicone model was set to 2.13 ± 0.08 MPa at room temperature, and the external size of the silicone block was set to 100 mm × 60 mm × 30 mm to prevent expansion of the silicone model. The expansion of the inner diameter in the circulation was less than 0.3% (0.018 mm). The circuit was filled with a mixed solution of glycerin and fluorescent particles (FLUOSTAR, EBM Co., Ltd, Tokyo, Japan) and the refractive index was adjusted to that of the silicone models. The viscosity of working fluid was adjusted to 4.80 ± 0.02 mPa s at 37 °C, which was the average value of human whole blood obtained in this study. Blood flow rate and pressure were regulated to the same conditions as in the blood circulation test. An Nd:YAG laser beam (DS20-527, Photonics Industries, Bohemia, NY, USA) was used to irradiate the interface between the connectors and the silicone model. The PIV images were taken using a high-speed camera (VC13-0192, Imager Pro XL, LaVision, Goettingen, Germany) through an optical filter with a cutoff frequency of 550 nm (CVI Melles Griot, NM, USA). Pixel size was 0.025 mm and frame speed was 400 frames/s.

### Statistical analysis

Statistical analysis was performed using SPSS (version 21, IBM, Tokyo, Japan). Changes in the platelet count and hematocrit before and after testing were compared using Student’s *t* test.

The average differences in the thrombus formation areas between the connector inlet and outlet after circulation were compared using Student’s *t* test.

## Results

### Blood conditions

The activated clotting time at the beginning of the test was 161 ± 20 s. The activated clotting times after 1 h of blood circulation for the connector without tapers and connector with tapers were 161 ± 20 and 160 ± 18 s, respectively. The platelet count at the beginning of the test was 19.7 ± 2.8 × 10^4^/μL. Platelet counts after 1 h of blood circulation for the connector without and with tapers were 16.6 ± 3.5 × 10^4^ and 16.0 ± 3.8 × 10^4^/μL, respectively. The platelet count after 60 min of blood circulation decreased regardless of the connector design. There were no significant differences in the platelet count between the connectors without and with tapers (*p* = 0.77). The hematocrit at the beginning of the test was 42 ± 2%. The hematocrit after 1 h of blood circulation in the circuits incorporating the connector without and with tapes was 41 ± 2 and 41 ± 2%, respectively. There were no differences in the hematocrit between connectors without and with tapers (*p* = 0.63), and before and after 1 h circulation.

Blood pH did not change before and after the 60 min of circulation (before: 7.34 ± 0.01, after: A 7.34 ± 0.01; B 7.34 ± 0.01). Blood viscosity before circulation was 4.78 ± 0.17 mPa s (*n* = 6).

### Quantification of thrombus formation area

**Fig. 3:**
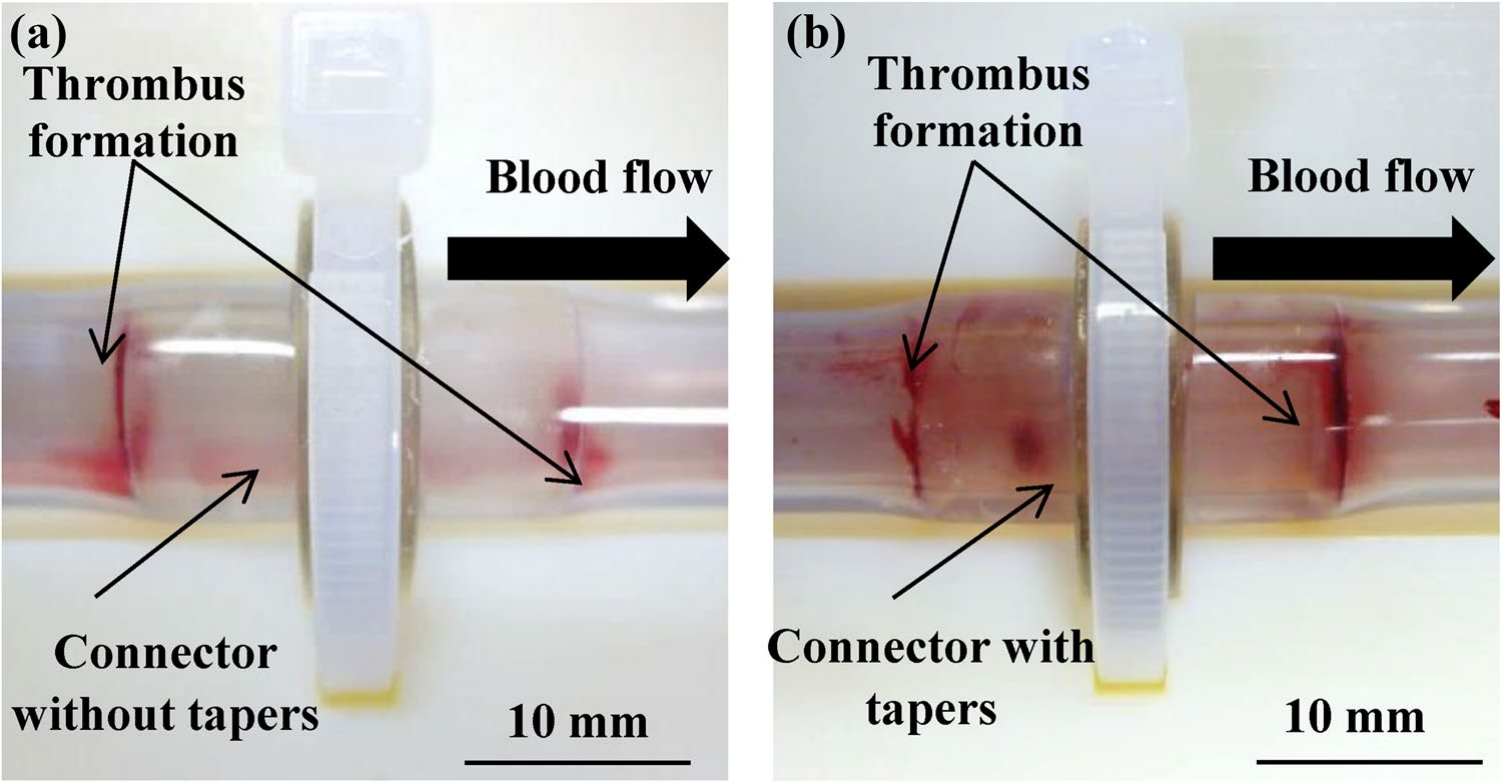
Observation of thrombus formation at the connector interfaces. The lumen was washed 3 times with phosphate-buffered saline after 1 h of blood circulation. *Black arrows* indicate the direction of blood flow. **a** Connector without tapers. **b** Connector with tapers

The interface between the connector and the tube during 60 min of blood circulation was observed after a careful rinse with phosphate-buffered saline (Fig. 3).

**Fig. 4:**
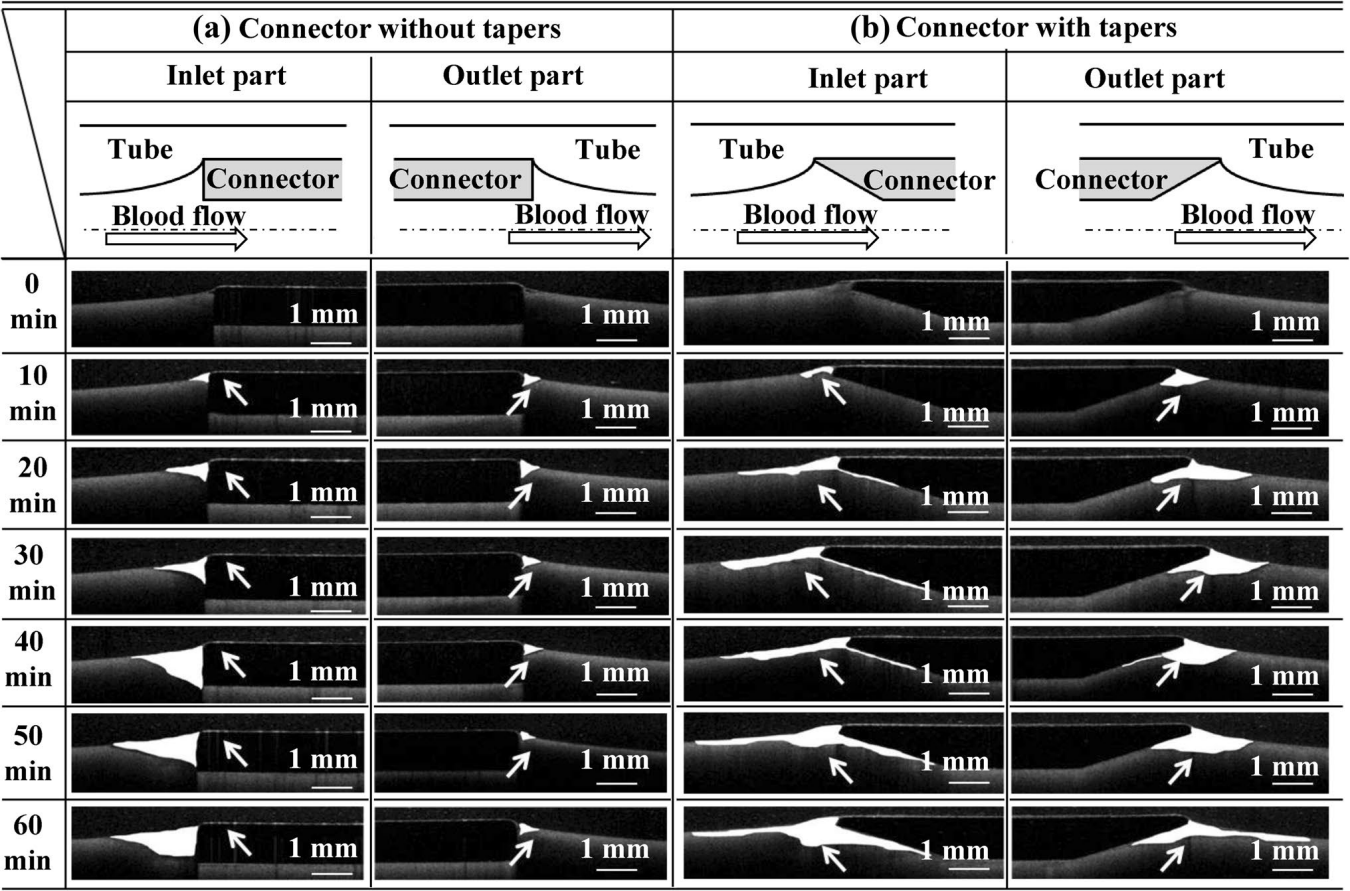
Sequential changes in the thrombus formation sites at the interface between the connector and the tube. **a** Thrombus formation at the interface between connector without tapers and tube. **b** Thrombus formation at the interface between connector with tapers and tube. The blood circulation time is shown in the *left* column

**Fig. 5:**
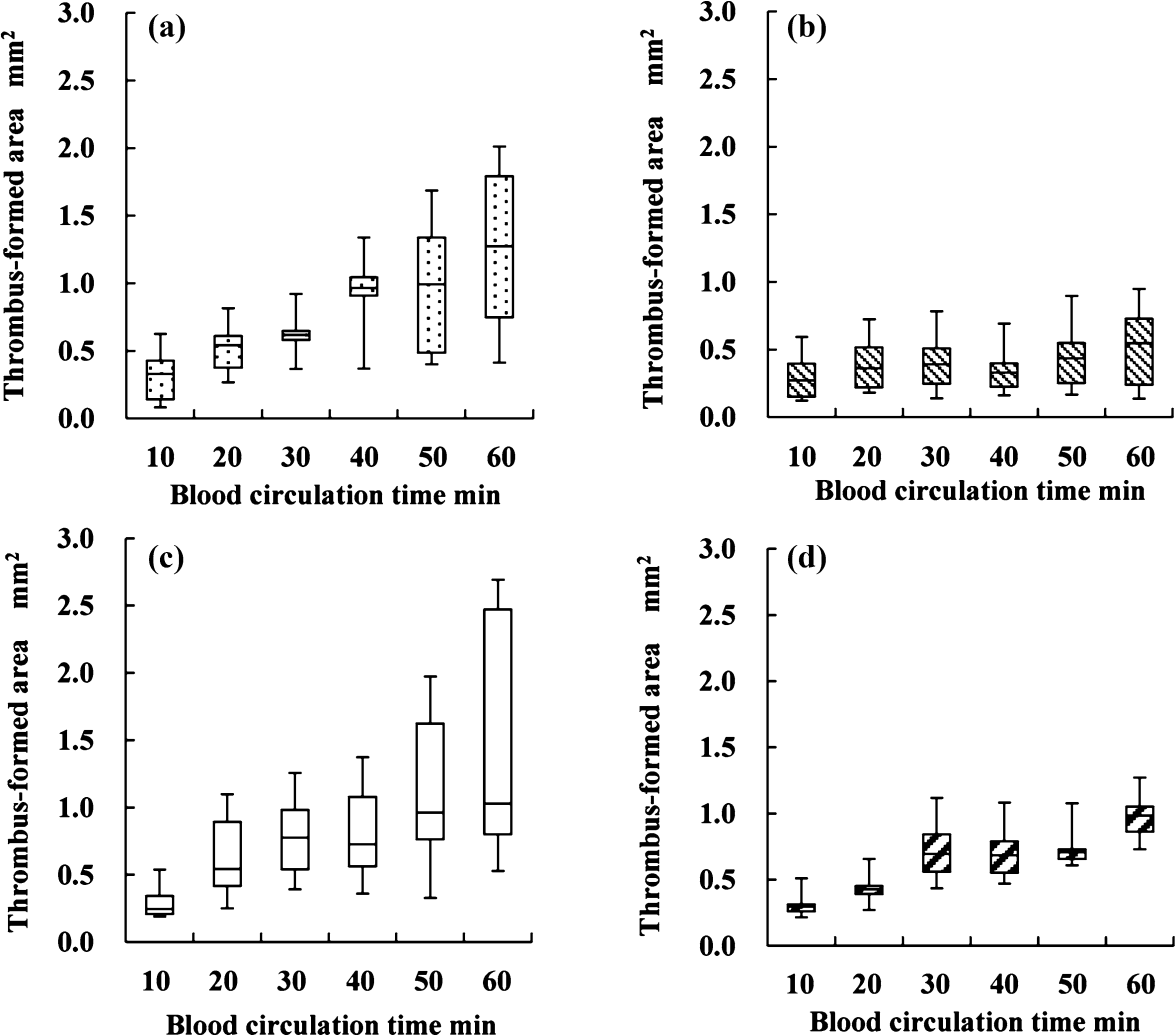
Quantitative changes in sequential thrombus formation area measured with optical coherence tomography. **a** At the inlet interface of the connector without tapers. **b** At the outlet interface of the connector without tapers. **c** At the inlet interface of the connector with tapers. **d** At the outlet interface of the connector with tapers

**Fig. 6:**
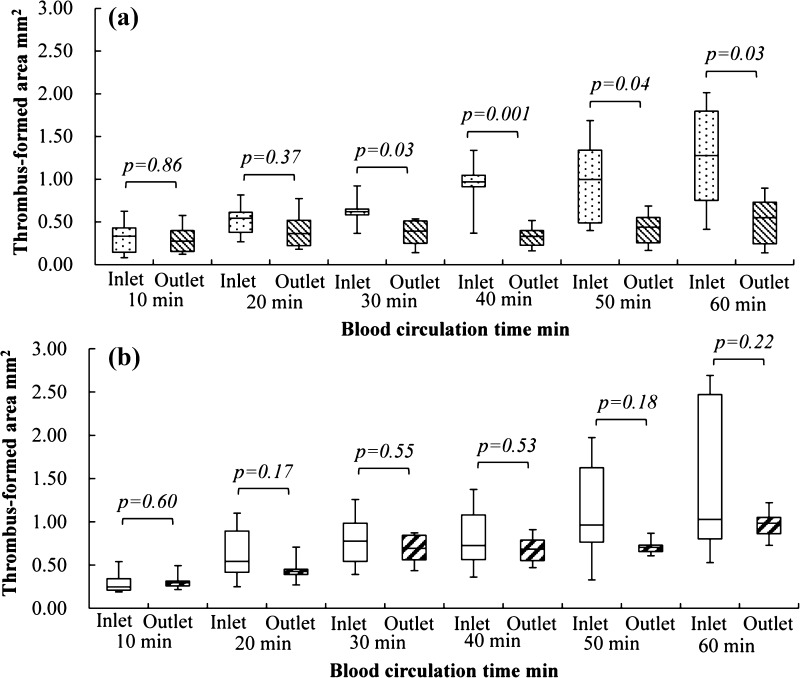
Comparison of thrombus formation areas between the connectors inlet and outlet. **a** Thrombus formation area at the inlet and outlet of the connector without tapers. **b** Thrombus formation area at the inlet and outlet of the connector with tapers

The sites of thrombus formation during 10–60 min of circulation were visualized at 10-min intervals (Fig. 4) using OCT. The sequential changes in the thrombus formation area at the inlet interface between the connector without tapers and the tube as well as at the outlet interface between the connector without tapers and the tube were quantified (Fig. 5a, b). The thrombus formation area at the inlet interface between the connector without tapers and the tube increased from 0.32 ± 0.21 to 1.25 ± 0.66 mm^2^ during circulation from 10 to 60 min (Fig. 5a). On the other hand, the thrombus formation area increased less at the outlet interface between the connector without tapers and the tube (from 0.30 ± 0.18 mm^2^ at 10 min to 0.51 ± 0.31 mm^2^ at 60 min) (Fig. 5b). Compared with the outlet interface, a larger amount of thrombus formed at the inlet interface of the connector without tapers in 30 min or more of circulation (*p* < 0.05) (Fig. 6a).

A similar thrombus formation process was found for the interface between the connector with tapers and the tube. The thrombus formation area increased from 0.27 ± 0.08 to 1.55 ± 1.12 mm^2^ at the inlet interface during circulation from 10 to 60 min (Fig. 5c), and slightly increased from 0.29 ± 0.06 to 0.94 ± 0.14 mm^2^ during circulation from 10 to 60 min at the outlet interface (Fig. 5d). The amount of thrombus was not significantly different between the inlet and outlet interfaces for the connectors with tapers (Fig. 6b).

**Fig. 7:**
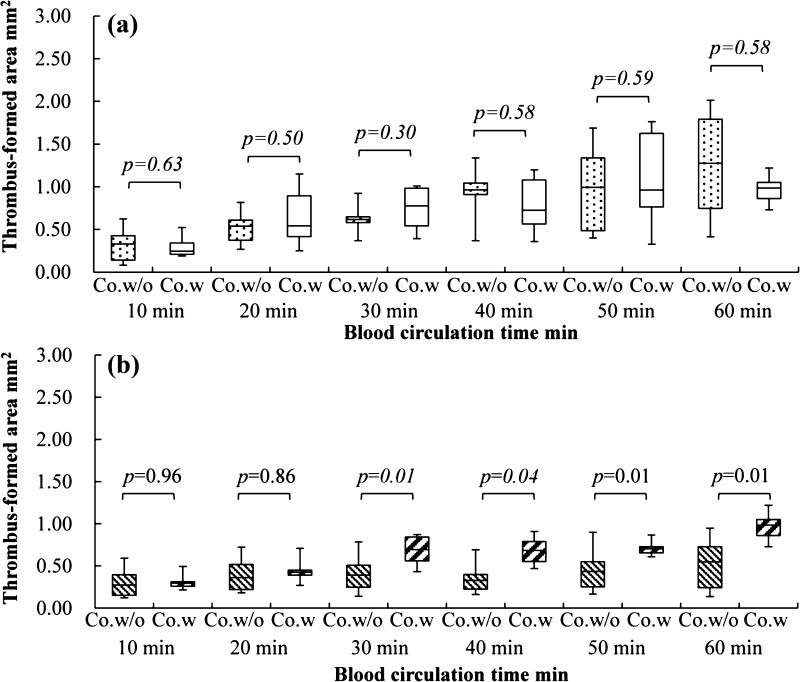
Comparison of the thrombus formation area between the connectors without and with tapers at the inlet/outlet interfaces. **a** Thrombus formation area at the inlet of connector without tapers (Co.w/o) and connector with tapers (Co.w). **b** Thrombus formation area at the outlet of connector without tapers (Co.w/o) and connector with tapers (Co.w)

There was no significant difference in the thrombus formation area between the connectors without and with tapers at the inlet interface during 60 min of circulation (*p* = 0.72) (Fig. 7a). On the contrary, a larger amount of thrombus formed at the outlet interface for the connector with tapers than for the connector without tapers in not less than 30 min of circulation (*p* < 0.05) (Fig. 7b).

### Blood flow stream and differences in flow velocity

**Fig. 8:**
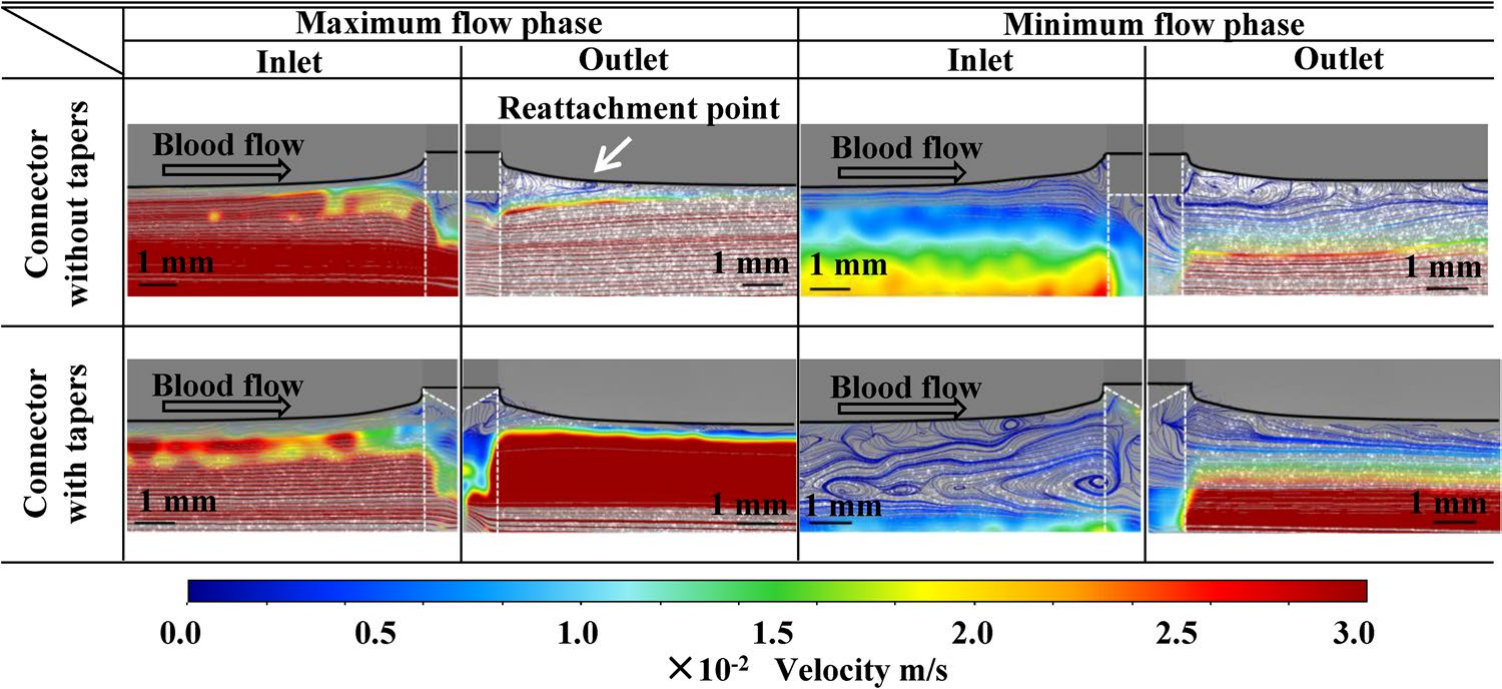
Flow streams in the vicinity of the connector without tapers and connector with tapers. *White dotted line* indicates the area of connectors

Flow velocity distribution at the interface between the connector and tube was examined using particle imaging velocimetry. Flow distributions at the maximum and minimum flow phases during pulsatile flow were observed at the connectors without and with tapers, respectively (Fig. 8).

A larger stagnant flow region was observed at the inlet of the connector with tapers in the minimal flow phase.

A reattachment point of flow was observed approximately 1.5 mm downstream from the outlet junction of the connector and tube for the connector without tapers (Fig. 8, arrow).

## Discussion

To our knowledge, this is the first report to quantitatively show the thrombus growth process using non-invasive OCT. The optical scattering properties from blood change by blood cells components and plasma, flowing conditions, and degree of blood coagulation [19]. At the site of thrombus formation, erythrocytes are trapped by adhered platelets and fibrin network [14]. Therefore, the time-dependent change in the scattered light intensity from erythrocytes is reduced at the site of thrombus formation compared with that in circulating flow. Changes in the optical scattering intensity from erythrocytes at the site of thrombus formation were distinctly lower than those in circulating flow.

In this study, we clearly demonstrated a sequential thrombus formation process at the interface between the connectors and the tube. Initiation and growth of thrombus were associated with local hemodynamics. Thrombus was initially formed at the interface between the connector tip and the tube. Geometries of thrombus growth were different between the 2 connectors and between the inlet and outlet (Fig. 4). Growth of thrombus was observed at the interface between the connectors and tubes over time during 60 min of circulation, except at the interface between the connector without tapers outlet and the tube. At the sites of thrombus formation and thrombus growth in 60 min blood circulation tests, flow separation or recirculating flow was observed in the flow visualization study. These data indicated the impact of local flow for managing thrombus growth. At the outlet interface between the connector without tapers and the tube, thrombus propagation length from the connector edge toward the flow downstream was comparable at 10 and 60 min (0.55 ± 0.35 vs. 0.51 ± 0.32 mm, *p* = 0.81). Flow analysis using particle image velocimetry showed the presence of a flow reattachment point 1.5 mm downstream from the connector edge. These results suggest that the flow reattachment point inhibits downstream thrombus growth. These data implied that a connector without taper at the outlet part may reduce thrombus-related complication by preventing detachment of thrombus formed at the interface between connectors and tubes. These findings encourage us to further research an ideal connector design and ideal design of the interface between connectors and tubes.

In this study, platelet count was decreased after 60 min blood circulation each in the circuit with the connector without tapers and with tapers. After the circulation, thrombus was observed only at the interface between the connectors and tubes in the whole circuits. The sum of thrombus formed area after the circulation was comparable between the interfaces of connector without and with tapers. These data suggest that platelet was mainly consumed for thrombus formation at the interfaces of the connectors and tubes.

The method presented here would be useful to investigate influences of the softness of the tube and a minute gap of the diameter between the tubes and connectors, which induce changes in the shape of the interface between the connector and tube, on thrombus formation and thrombus propagation at the interfaces. Using the methods presented here, the influence of the 3-dimensional geometry of the connecter and the subsequent geometry of the interface between the connector and the tube on thrombus growth can be thoroughly assessed, with the aim of developing a novel connector and interface to inhibit the detachment of thrombus.

## Conclusion

We quantitatively demonstrated the sequential thrombus formation process at the interface between the connectors and the tube during pulsatile flow of human blood using non-invasive OCT. This study clearly showed that the initiation and growth of thrombus were associated with local hemodynamics. The methods presented here may contribute to developing a novel connector to inhibit the detachment of thrombus formed at the interface between the connector and the tube, thus reducing thrombus-related complications.
